# The Biochemical Properties of Manganese in Plants

**DOI:** 10.3390/plants8100381

**Published:** 2019-09-27

**Authors:** Sidsel Birkelund Schmidt, Søren Husted

**Affiliations:** Department of Plant and Environmental Sciences, Faculty of Science, University of Copenhagen, 1871 Frederiksberg C., Denmark; shu@plen.ku.dk

**Keywords:** Manganese, enzyme, metalloenzyme, database, catalysis, photosystem II, Mn cluster, superoxide dismutase, oxalate oxidase

## Abstract

Manganese (Mn) is an essential micronutrient with many functional roles in plant metabolism. Manganese acts as an activator and co-factor of hundreds of metalloenzymes in plants. Because of its ability to readily change oxidation state in biological systems, Mn plays and important role in a broad range of enzyme-catalyzed reactions, including redox reactions, phosphorylation, decarboxylation, and hydrolysis. Manganese(II) is the prevalent oxidation state of Mn in plants and exhibits fast ligand exchange kinetics, which means that Mn can often be substituted by other metal ions, such as Mg(II), which has similar ion characteristics and requirements to the ligand environment of the metal binding sites. Knowledge of the molecular mechanisms catalyzed by Mn and regulation of Mn insertion into the active site of Mn-dependent enzymes, in the presence of other metals, is gradually evolving. This review presents an overview of the chemistry and biochemistry of Mn in plants, including an updated list of known Mn-dependent enzymes, together with enzymes where Mn has been shown to exchange with other metal ions. Furthermore, the current knowledge of the structure and functional role of the three most well characterized Mn-containing metalloenzymes in plants; the oxygen evolving complex of photosystem II, Mn superoxide dismutase, and oxalate oxidase is summarized.

## 1. Introduction—Manganese as an Essential Plant Nutrient

Manganese (Mn) is an essential micronutrient in most organisms. More than 200 years ago, the presence of Mn in leaf tissue of plants was experimentally documented for the first time in the ash of several vegetable species [1]. During the following 100 years, Mn was found in most important crops, but it was not until the early 1900s that Mn was documented as being required for plant growth and development [2,3]. The probable role of Mn in oxygenic photosynthesis was demonstrated for the first time by Pirson and co-workers, who recognized that plants and algae are unable to release O_2_ in the absence of Mn in their growth medium [4,5]. Later, the redox-dependent function of oxygen evolution was discovered by Kok et al. [6]. However, the biological role of Mn in catalyzing oxygen evolution remained unclear until Spector and Winget [7] managed to isolate a catalytically active Mn-containing protein complex from spinach, providing evidence that Mn is an integral and essential metal cofactor in the oxygen evolving complex (OEC) of higher plants.

Manganese biogeochemistry in soils is complex, because Mn exists in three oxidation states, Mn(II), Mn(III), and Mn(IV) in the soil solution. Only the divalent form (Mn^2+^) is available for plant uptake, as Mn(III) is unstable and Mn(IV) forms highly insoluble oxides and precipitates. Although Mn is abundant in most soils, particular soil pH and redox conditions greatly influence the concentration of Mn^2+^ in soils, with a high pH reducing its availability through MnO_2_ formation, whereas reducing O_2_ in the soil air by soil compaction or flooding, increases the Mn^2+^ concentration [8]. In the field, Mn deficiency therefore commonly appears patchy due to an uneven distribution of the soil conditions favoring Mn oxidation or reduction. Manganese deficiency is a widespread problem, particularly in dry, calcareous, and sandy soils worldwide [9,10,11,12,13,14,15]. Symptoms of the deficiency develop as interveinal chlorosis in newly emerged leaves, while prolonged deficiency causes leaf necrosis, which appears as brown spots between veins in older leaves [8]. In problematic regions, Mn deficiency significantly reduces crop yields, and can even cause complete crop loss in severe winters [10].

Manganese is a metal co-factor for approximately 6% of all known metalloenzymes [16]. A relatively large number of enzymes are activated by Mn^2+^, but only a few metalloenzymes are currently known to have an absolute and non-replaceable requirement for Mn to become catalytically active [17]. In plants, the most well studied and described Mn-dependent enzymes include the Mn superoxide dismutase (Mn-SOD), oxalate oxidase (OxOx), and the Mn cluster in photosystem II (PSII), catalyzing the split of water, which provides the necessary electrons for driving photosynthesis. Thus, a lack of available Mn has negative impacts on plant growth and development by precluding the functional roles associated with these key-enzymes, such as scavenging of reactive oxygen species, seed germination, and fungal protection as well as photosynthesis in plants.

## 2. The Biological Chemistry of Manganese in Plants

**Figure 1:**
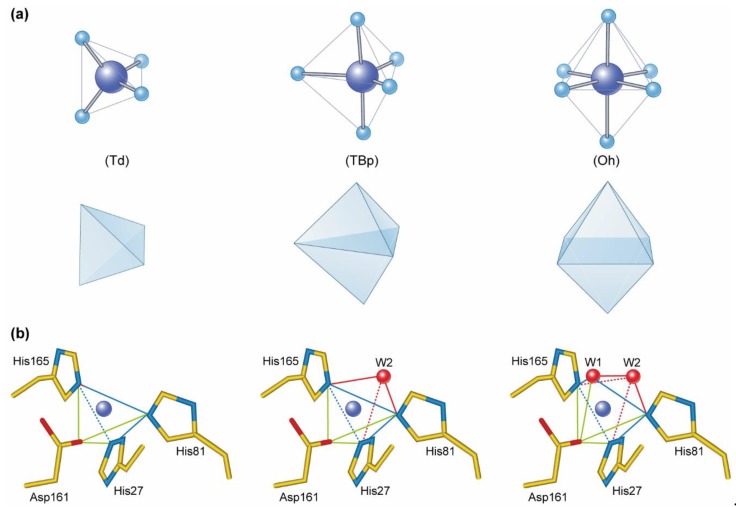
(**a**) The three dominating coordination geometries and coordination numbers observed in Mn (purple sphere) containing metalloproteins are tetrahedral (Td, *n* = 4); trigonal bipyramidal (TBp, *n* = 5) and octahedral (Oh, *n* = 6). (**b**) The active site of Mn-SOD in the presence of Mn (purple sphere), with and without water molecules (W1 and W2, red spheres) in the coordinating sphere. Three His residues (His 165, 27, and 81), one Asp (161) coordinates Mn, and the involvement of 0, 1, or 2 water molecules determines the geometry of the Mn complex at the active site. Zero water produces Td, binding of W1 yields TBp, and binding of W1 and W2 produces the Oh geometry. It has been speculated that the geometry of the active sites changes during scavenging of reactive oxygen species [22].

Manganese is a first-row transition element found in nature as the stable isotope ^55^Mn. Manganese exists in the oxidation states +2, +3, +4, +6, and +7, but the +2 (Mn(II)) oxidation state is most prevalent in biological systems. However, extensive redox cycling between the oxidation states +2, +3, and +4 occurs in the rhizosphere, in plant Mn metalloenzymes, such as Mn-SOD [18], OxOx (Whittaker et al., 2007), and in the OEC of photosystem II (PS II) [6]. The most common coordination geometries of Mn in proteins is octahedral (Oh, coordination number, *n* = 6), square pyramidal and trigonal bipyramidal (TBp, *n* = 5) and tetrahedral (Th, *n* = 4) (Figure 1). Manganese ions exhibit fast ligand exchange kinetics, which means they can easily be replaced by other divalent metal ions, including Mg, Ca, Fe, Co, Cu, and Zn. The selection of metal centers in metalloenzymes has been influenced by the availability of metals through geological time, as the readiness of specific metals appears to be the driving force behind most of enzyme evolution [19]. Before the emergence of oxygen-evolving photosynthesis, the earth existed in a strong reductive environment, with the ancient oceans having large sulfur contents that affected metal solubility with Fe and Mn dissolving and, Cu and Zn precipitating under these conditions. In contrast, under current oxygenic conditions the bioavailability of these metals are completely reversed, with the affinity for divalent metal ions to the exchange of complexed water (H_2_O) for any other ligand within a metal complex given by the Irving–Williams series [20]: Mg^2+^ < Mn^2+^ < Fe^2+^ < Co^2+^ <Ni^2+^ < Cu^2+^ > Zn^2+^.This states that Mn^2+^ is a relative weak binding metal in proteins. 

Metal ions are characterized as Lewis acids because they can accept a pair of electrons (a lone pair) from a donor molecule and form a coordinate covalent bond. Ligands are defined as Lewis bases as they donate lone pairs to the metal ion when a complex is formed. The strength of a Lewis acid is largely determined by the charge density, i.e., the higher the valence and the smaller the ion radius, the stronger the acidity of a given metal ion. Ions with high charge density in combination with a low polarizability, which is a measure of how easily an electron cloud is distorted by an electric field, are designated as hard acids. Hard acids tend to form strong complexes with hard bases, whereas soft acids form strong bonds with soft bases [21]. This trend is named the “HSAB” rule: “the hard–soft acid–base rule” and is a very convenient measure of the affinity between metal ions and ligands (Table 1) [21].

Among the biological relevant transition elements, Mn^2+^ is considered a “hard” to “borderline” ion, i.e., an ion with a large charge density and a low polarizability. As a consequence, Mn prefers coordination with hard ligands such as the negatively charged oxygen atoms in the carboxylate groups of aspartate (Asp) and glutamate (Glu), as well as the polar oxygen atoms within carbonyl groups of asparagine (Asn), glutamine (Gln) and water molecules (H_2_O). The borderline aromatic N containing imidazole ring of histidine (His) is another important ligand for Mn^2+^. In contrast, the sulfur (S) containing amino acids cysteine and methionine are soft ligands and are consequently less likely to coordinate with Mn^2+^. Up until now there is no identification of Mn metalloenzymes which contain a Mn-S coordination sphere [25], but several synthetic Mn complexes with both oxygen (O), nitrogen (N), and S ligands have been characterized [18].

## 3. Manganese in Metalloenzymes

**Figure 2:**
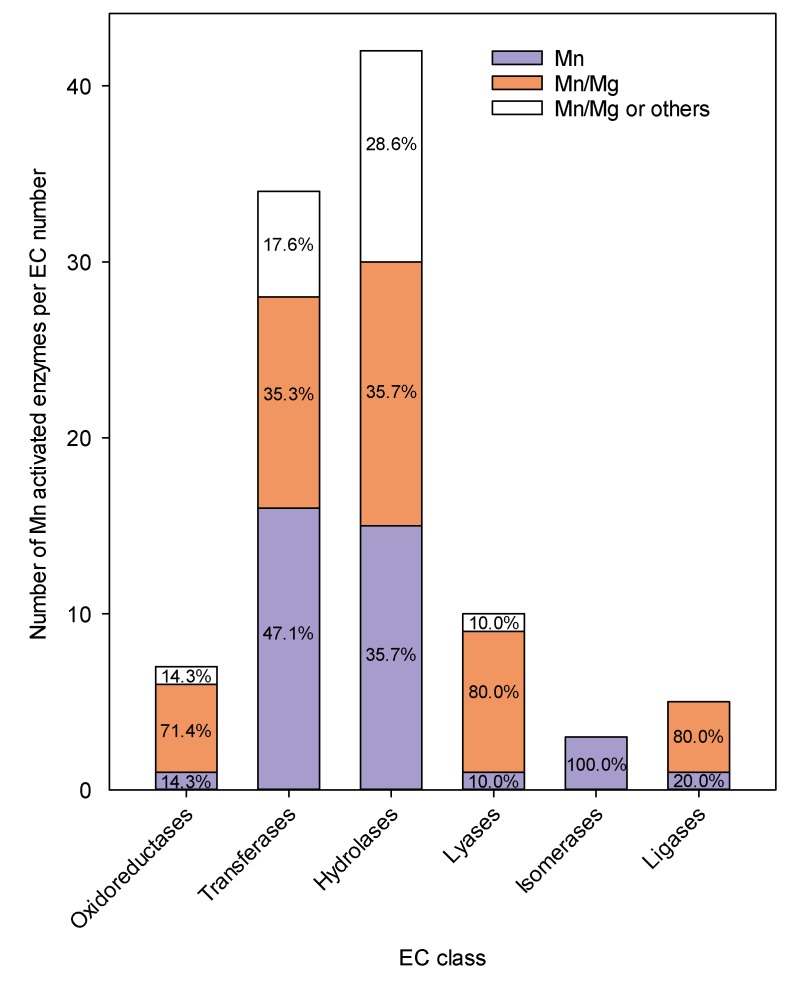
Distribution of Mn activated enzymes across the six Enzyme Commission (EC) classes together with the relative frequencies (%) of the coordinated metal ion for each EC class. A total of 101distinct Mn activated enzymes is described in the UniProt Knowledgebase for *Arabidopsis thaliana*, when filtered by reviewed records, accessible cofactor information, and protein name. Of these enzymes, 37 exclusively contain Mn as a cofactor, 44 enzymes are activated by either Mn or Mg, while 20 enzymes are reported to coordinate with Mn, Mg, or other divalent metals (Ca, Zn, Fe, Co, Ni, Cu).

Manganese has two major functions in enzymes: (i) as a Lewis acid, for which the properties of Mn are similar to Mg, Co and Zn and (ii) as an oxidation catalyst, for which it can be compared with properties similar to that of other redox active metal ions, such as Fe and Cu (Table 1). In Arabidopsis, 101 identified enzymes contain Mn in the active site, but only one third of them appear to have an absolute requirement for Mn (The UniProt Knowledgebase (http://www.uniprot.org), accessed 2 July 2019) (Figure 2). For the majority of Mn containing metalloenzymes, Mn is interchangeable with other divalent metal cation. Manganese (II) has a radius of approximately 0.78 Å, somewhat larger than that of magnesium (Mg, 0.66 Å), but smaller than that of calcium (Ca, 0.99 Å), and almost the same as that of zinc (Zn, 0.72 Å), copper (Cu, 0.72 Å), and cobalt (Co, 0.74 Å). Considering the similar sizes, charges and HSAB classification of Mn and the other transition metals listed in Table 1, they might be expected to substitute for each other in proteins and in biochemical reactions. However, this is rarely the case, as the biochemical behavior of Mn^2+^ resembles that of Mg^2+^ more than e.g., Zn^2+^ and the remaining transition elements listed (Table 1).

Manganese and Mg ions share a number of chemical and biochemical characteristics: The ionic radii of Mg^2+^ (0.66 Å) and Mn^2+^ (0.67 Å) are almost identical in the low spin state (bound state), where the metal ions have as many electrons paired as possible. The orbital composition of Mn is very different from that of Mg because it includes *d* orbitals. A shift from a low spin state to a high spin state (free ions, Table 1), does not influence the ionic radius of Mg^2+^, which remains at 0.66 Å, whereas that of Mn^2+^ substantially increases to 0.78 Å [26]. These metals, characterized as hard (Mg) to hard-borderline (Mn) metal ions, have the same ability to form divalent cations, and may form complexes of the same geometry using oxygen ligands (Table 1). Thus, generally few differences exist between Mg and Mn coordination spheres responsible for the cation specificity. Khrustalev et al. [27] investigated several hundred Mn and Mg metalloproteins and found that Mg and Mn are typically bound to oxygen in the carboxylate groups of Asp and Glu. Histidine (His) residues appear underrepresented in Asp and Glu rich pockets binding Mg^2+^, while His groups are overrepresented in Asp and Glu pockets binding Mn^2+^. When replacing Mg in the active site with divalent Mn, the catalytic activity of the enzyme is often maintained [28]. In contrast, the enzyme functionality is often lost when Mg exchanged with Mn in a Mn-dependent enzyme. The exact reason for this striking difference between Mg and Mn specificity remains to be resolved [29]. Due to the similarities of the coordination spheres for Mn and Mg and due to their low rank in the Irving–Williams series, the potential risk of mismetallation in Mn and Mg metalloproteins is high. In the plant cell, the risk of mismetallation is reduced by cellular compartmentation and a tight homeostatic regulation. In leaf tissue, most Mn and Mg is sequestered in the vacuoles, and allocation to the chloroplasts, golgi and mitochondria, where both metals serve key functions, is tightly regulated at the Mn and Mg transporter level [30,31].

Across the 6 enzyme classes, 44% of all Mn containing metalloenzymes can bind Mg with different Mn/Mg stoichiometries (Figure 2). It is important to note that information about metal binding in metalloproteins must be viewed with caution, as evidence is most often based on in vitro experiments, where the metal might be lost or exchanged during the extraction and purification process. It is therefore likely that Mg (an essential macronutrient) dependent enzymes are overrepresented in Figure 2, as Mg is typically found in 100-fold excess relative to Mn (being an essential micronutrient) in homogenized plant tissue and in specific cell compartments, including the cytosol and chloroplasts [32,33]. In fact, only very few of the currently known Mn-bound enzymes have been experimentally validated.

Substitution of Mg with Mn typically changes the catalytic rate of the enzyme and in many cases it also changes the functional role of the enzyme [34]. Rubisco (Ribulose-1,5-bisphosphate carboxylase/oxygenase) illustrates well how differential binding of Mg and Mn changes the catalytic rate and the substrate preference of a protein (“one protein, two enzymes”). Rubisco is the most abundant protein in plants and catalyzes the carboxylation reaction by fixing atmospheric CO_2_ in the dark reactions of photosynthesis together with the oxygenation reaction fixing O_2_ during photorespiration. When Rubisco binds to Mg^2+^, carboxylation is favored and proceeds 4 to 11 times faster than oxygenation, but when Rubisco binds to Mn^2+^ carboxylation and oxygenation occur at similar rates [35]. When Mn is bound to Rubisco it participates in the catalytic process of ribulosebiphosphate (RuBP) oxygenation and becomes reduced with every oxygenation event [34]. However, preference is clearly given to binding of Mg as the oxygenating capacity of Mn-activated Rubisco is so large that photosynthesis supported by this catalyst would be incapable of sustaining a positive carbon balance in the Earth atmosphere. Kinetic studies have revealed that if Mn was the only metal ion to coordinate with Rubisco then the CO_2_ compensation point in the mesophyll would need to rise 30-fold (>1500 ppmv) to support photosynthesis. Thus, preference of Mg binding to Rubisco is therefore a prerequisite for autotropic life on the planet [34], despite this the regulation of metal insertion (Mg vs. Mn) is not fully understood.

## 4. Metallation of Manganese Containing Metalloenzymes

Manganese transporters are essential for uptake, distribution, and storage in the cell, with several different transporter families documented to be implicated in Mn transport in plants [36]. However, the molecular mechanism used by cells to avoid mis-metallation of enzymes in the presence of competitive metals remains to be fully resolved. One way to ensure that the correct metal is inserted into the active site of a metalloenzyme is to exploit delivery proteins, also known as chaperones. Metallochaperones play an important role in delivering metal ions to apo-proteins by ensuring correct insertion into the active site of the protein in the multi-ionic environment of the cell, where competing ions may exist in much larger concentrations and with greater affinities towards potential ligands.

The current knowledge of Mn chaperones is mostly obtained from studies in cyanobacteria, and little is known about Mn chaperones in higher plants. Synechocystis PratA (for processing-associated tetratricopeptide (TPR) protein) functions as an assembly factor required for efficient delivery of Mn^2+^ to the reaction core of PSII in vivo, during the early steps of thylakoid biogenesis [37]. PratA specifically binds Mn and is assumed to preload the PSII core D1 protein with Mn^2+^ in the early stages of PSII assembly. In PratA deletion mutants, the Mn^2+^ loading is reduced by almost 9-fold relative to the wildtype. The Mn binding constant for PratA is much smaller than that observed for other metalloenzymes in which Mn is bound specifically and irreversibly (e.g., Mn-SOD) [38]. This underlines the more transient binding of Mn in PratA allowing it to act as a metallochaperone [37]. 

In land plants it is speculated that the extrinsic protein PsbP plays a role similar to PratA, by acting as a chaperone for insertion of Mn into the PSII reaction core, where it subsequently, stabilizes and shields the Mn cluster of PSII in association with PsbO and PsbQ, [39,40]. High-resolution crystal structures of PsbP purified from spinach and *Zea mays* have revealed that two Mn ions are bound within the protein [41]. The first Mn ion (Mn1) is bound in a positively charged pocket coordinated by Cl^−^ and His and Asp groups, and the second Mn (Mn2) in a negatively charged pocket coordinated by Asp and water. Based on protein structural analysis it was proposed that Mn1 represents a high affinity binding site, whereas Mn2 is bound with low affinity. The Mn2 coordination site is believed to be responsible for the proposed chaperone action, as Mn is more loosely bound and thereby able to donate Mn ions to the reaction core of PSII [41]. As supporting evidence, a marked conformational change of PsbP is observed when Mn2 is lost; leading Ido et al. [42] to speculate that the functional interaction of PsbP with PSII is controlled in a pH-dependent manner.

There are only few cases where Mn-dependent metalloenzymes are known to effectively use metal ions other than Mn to catalyze a reaction [43]. An example is Fe/Mn substitution in cambialistic SODs, which are able to retain a similar activity with either Fe or Mn in the active site [44] (for more details, see section ‘Mn-dependent superoxide dismutase’). However, for the majority of enzymes, Mn substitution with Fe or any other metal ion is lethal to the enzymatic activity. In addition to chaperones, compartmentalization is another strategy for correct metallation by keeping competitive metals out of the wrong nascent proteins. Tottey et al. [45] demonstrated how the most abundant Cu protein, Cu-cupin A (CucA), and the most abundant Mn protein, Mn-cupin A (MncA), from the periplasm of cyanobacterium both bind their respective metal via identical ligands in a cupin fold. Notably, the cupin prefers Cu to Mn in vitro consistent with the Irving–Williams series. Even so, Mn manages to coordinate with cupin in a multi-ionic environment when present in a 10^4^-fold excess relative to Cu. When Mn was bound to the cupin site it possessed high thermodynamic stability and no ligand exchange with Cu^2+^ or Zn^2+^ was observed. This finding indicates that the concentration of Mn in the cytoplasm, where coordination and folding happens, is able to override the affinity of the coordinating sphere of the protein. MncA and CucA have Tat (twin-arginine translocation pathway) and Sec (secretion pathway) signal peptides, respectively, revealing different import pathways of the two proteins into the periplasm. The Tat pathway allows import of folded metallated proteins and thus enables MncA to fold in the cytoplasm where most Cu and Zn ions are tightly bound in complexes and free activity of these ions are extremely low, whereas Mn^2+^ is found in µM concentrations. In contrast, Cu outcompetes Mn in the periplasm leading to a preferential CucA folding, revealing a compartmentalization strategy, whereby the cellular compartment in which the protein folds overrides its binding preference in order to control the metal cofactor incorporation.

## 5. Manganese at the Active Site of Water Oxidation in Photosystem II

**Figure 3:**
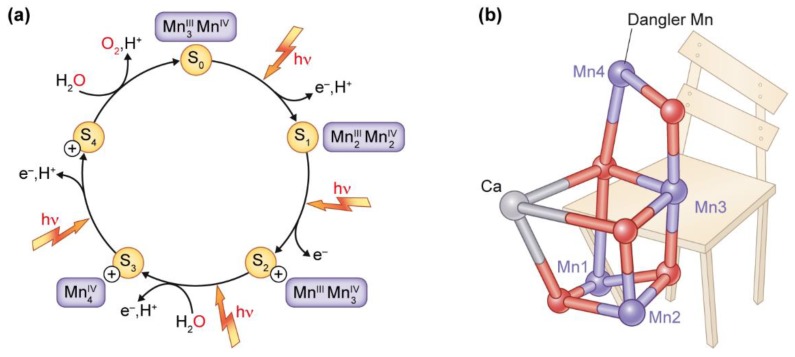
(**a**) The Kok cycle showing the steps of oxygen evolution in the oxygen evolving complex (OEC) of photosystem II (PSII) with details of the S_i_ states (S_0_–S_4_), the light induced oxidation steps of electron (e^−^) and proton (H^+^) release, together with the uptake of two molecules of H_2_O, which can be summarized to: $2H_{2}O\;\to \;4H^{+}+4{\;e}^{-}\;+O_{2}$. (**b**) The chemical structure shows the configuration and position of the metal atoms in the Mn4Ca cluster, resembling a distorted chair-like form. The distorted seat base is formed by three Mn and one Ca atoms in a cubane structure, and the back is formed by the fourth Mn (Mn4), the so-called dangler Mn, which lies outside the cubane, and has been proposed to act as the site of catalysis.

The most well studied Mn-containing enzyme is the Mn-cluster, also known as OEC, which is situated and surrounded by a protein matrix in photosystem II. PSII performs a series of electron transfer reactions using solar energy. The light-induced water oxidation requires four electrons and four protons to be stripped from two water molecules. This necessitates four sequential oxidation events, which are catalyzed by the Mn-cluster of the OEC that cycles through different redox states, known as S*_i_* states (*i* = 0–4) (Figure 3a). The unique redox chemistry of Mn (i.e., Mn can carry five charges from Mn(II) to Mn(VII)) makes it an ideal element for building the OEC, in which accumulation of four charges is needed to oxidize water molecules into molecular oxygen. Each oxidation step removes one electron, and Kok et al. [6] proposed this as the explanation for the observed oscillation of the oxygen evolution pattern, shown by Joliot’s experiments with saturating light flashes being used to power the photosynthetic process [46,47].

The structural and functional details of the intact Mn_4_CaO_5_ cluster were not revealed until 2001, where Zouni et al. [48] determined the three-dimensional structure of the OEC at 3.8 Å resolution. Subsequently, Ferreira and co-workers suggested that the structure of the OEC is a Mn_3_Ca cubane structure [49], with the configuration of the metals inside the cluster resembling a distorted chair-like form (Figure 3b). The distorted seat base of the chair is formed by three Mn, one Ca, and four oxygen atoms, and the back of the chair is formed by the last Mn atom, the so-called dangler Mn, which lifts out the oxygen from its central position in the cluster (Figure 3b) [50]. This suggests that Mn4 has a more flexible location than the other metal atoms [51], and it might function as a gate for releasing protons from the Mn_4_CaO_5_ cluster [52,53]. In 2011, the oxo-bridges and exact distances among the individual metal atoms, along with the accurate orientation of the lateral amino acid binding residues, was revealed from the high-resolution structure of PSII at 1.9 Å produced by Umena et al. [54]. Despite great progress in the understanding of OEC in PSII, several challenges remain. One pertinent question is the structure of the elusive S4 state, and the understanding of the mechanism of O-O bond formation. Zhang and Sun [53] have proposed a novel mechanism where the dangler Mn4 acts as the site of catalysis, forming a Mn(VII)-dioxo intermediate following charge rearrangement of the Mn cluster in the S4 state, triggering O-O bond formation and oxygen evolution. Experimental studies enabling a possible stabilization and characterization of the S4 state would finally complete our knowledge of the Kok cycle [55].

The light-induced process in PSII is very efficient, with a quantum yield of over 90%. With a turnover time of OEC of only ≈2 ms and that of the whole PSII of ≈10 ms, OEC performs more than 10^5^ reaction cycle before it must be replaced [55,56]. The Mn_4_CaO_5_ cluster is assembled by the stepwise binding of Mn^2+^ ions, as well as light-driven photooxidation of Mn^2+^ to Mn^≥3+^ to the co-factor depleted PSII (apo-OEC), as the Mn atoms become coordinated within the ligation matrix of the active site, a process called photoactivation [57,58]. The D1 carboxylate ligand, Asp170, plays a pivotal role during the assembly process by forming a high affinity site for Mn binding, involved in the initial photooxidation of Mn^2+^ [59]. However, the major question about the photoactivation mechanism is where the initial Mn^2+^ binding site is in the apo-OEC. Among the five metal sites (Mn1–Mn4 and Ca) of the Mn_4_CaO_5_ cluster it was recently concluded that the Mn1 site, likely in equilibrium with the Mn2 site, is the site of initial Mn^2+^ binding, which is supported by the pH dependence of the dissociation constant of Mn^2+^ and the effect of the chemical modification of His-residues in apo-OEC. This suggests the involvement of a His side chain in the high affinity site of Mn^2+^ binding. Whether the Mn ions are recycled during PSII repair remains unknown.

Mn deficiency alters the macro-organization of PSII, as demonstrated by a pronounced decrease in the abundance of PSII-LHCII supercomplexes under increasing Mn deficiency [60]. Furthermore, the abundance of D1 together with the PSII membrane extrinsic proteins PsbP and PsbQ (but not PsbO) is significantly reduced under Mn deficiency [61,62,63].

## 6. Manganese-Dependent Superoxide Dismutase

Reactive oxygen species (ROS) are inevitable toxic byproducts of basic plant metabolism. ROS are extremely reactive with and harmful to all biomolecules, causing DNA and RNA damage, protein oxidation and lipid peroxidation (collectively referred to as oxidative stress) [64]. Plant cells have developed an anti-oxidative defense system employing the ROS scavenging enzyme superoxide dismutase (SOD) functioning in cellular adaptation to oxidative stress. SODs are a family of metalloenzymes, which catalyze the dismutation of superoxide radicals into molecular oxygen (O_2_) and hydrogen peroxide (H_2_O_2_).

In plant cells, ROS are primarily formed in chloroplasts, mitochondria and peroxisomes, and it is therefore crucial that SODs are present in these compartments for ROS detoxification. Based on the prosthetic metal used by SOD, being Cu, Fe, or Mn, plant SODs are classified into three groups: Fe-SOD, Mn-SOD, and Cu-Zn-SOD, which are localized to the chloroplast; mitochondria and peroxisomes; chloroplast and cytoplasm, respectively. While Fe and Cu are well-known for their reactivity with H_2_O_2_, which generates the highly reactive hydroxyl-radical via the so-called Fenton reaction, Mn is less prone to such chemistry owing to a greater reduction potential. The first Mn-SOD purified and biochemically characterized from a higher plant was the enzyme from pea leaves [65,66,67]. The regulation of Mn-SOD activity is far from trivial, as Mn deficiency has been reported to increase Mn-SOD activities in some studies but significantly inhibit the enzyme in others [68,69,70]. These contrasting findings are still under debate, but might be explained by differences in experimental setups and the plant species studied.

Plant Mn-SOD is typically found in mitochondria, but has also been detected in peroxisomes [71]. In higher plants, the molecular structure of Mn-SOD is homotetrameric, with a single Mn atom per subunit. The active site of Mn-SOD is highly conserved, and plant Mn-SODs share approximately 70% sequence similarity across a wide range of plant species [72,73]. In the resting state of Mn-SOD, Mn^3+^ is bound in a trigonal bipyramidal geometry (TBp, Figure 1) to the side chains of 3 His residues, one Asp residue, and one solvent ligand. The latter is thought to be a hydroxide ion when Mn-SOD is exerting the dismutase activity. This leaves an open coordination site on the metal, which allows the superoxide anion to bind to the Mn^3+^ center. The electron derived from superoxide oxidation is transferred to Mn^3+^, producing Mn^2+^-SOD and O_2_ [17,74]. The Mn-SOD dismutation mechanism involves the alternation of Mn-SOD between the oxidised (Mn^3+^) and less oxidised (Mn^2+^) state of Mn in the course of dismutating superoxide radicals [73,74]. The metal–ligand interaction within the active site of Mn-SOD follows the thermodynamics predicted by the HSAB rule [21]. The hydroxide and Asp ligands are hard bases, whereas His is a borderline base. Consequently, it follows the trend that hard-borderline acids like Mn^3+^ and Fe^3+^ should coordinate with the active site. It has been observed that the geometry of the coordination is the same, when the active site of Mn-SOD is bound to Fe^3+^ or Mn^3+^ [75]. They both demonstrate a five-coordinate geometry. However, it has been shown that Mn-SOD is only active when coordinated with Mn^3+^. When Mn^3+^ is bound to Mn-SOD the specific activity is more than 30 times higher than observed with Fe^3+^ coordination. Furthermore, it has been found that the redox potential of Fe^3+^ substituted Mn-SOD is too high to allow catalytic turnover of superoxides. Although Mn-SOD potentially binds both Mn^3+^ and Fe^3+^, the Fe substitution into a Mn-SOD protein matrix generally yields inactive proteins [76]. However, among the Fe/Mn SODs a sub-class of cambialistic SODs exists. These SODs have the capacity to utilize either Fe or Mn in their active site dependent on metal availability [77]. In plants, cambialistic SODs showing the same activity when either Fe or Mn is present was first reported by Chen et al. [44]. This observation implies the possible presence of an adaptation mechanism enabling a fine-tuning of the functional activity in response to different availabilities of metal micronutrients. A greater understanding of SODs with cambialistic properties will allow development of plants tolerant to multiple stresses to help satisfy the global growing demand of feed and food [78].

The correct insertion of Mn into Mn-SOD of Arabidopsis is likely to involve the AtMTM1-encoded protein targeted to the mitochondria in plant cells. The protein is regulated by the superoxide anion availability providing evidence for its involvement in activation of Mn-SOD [79]. Changes in SOD encoding gene transcript levels and SOD activity are generally regarded as indicators of the level of ROS production and oxidative stress [80]. A study in the green algae Chlamydomonas under Mn-deficient conditions showed a loss of Mn-SOD activity before that of PSII efficiency, suggesting a regulated intraorganellar supply of Mn to support PSII function in preference to Mn-SOD function in the mitochondria [81]. Thus, prolonged Mn-deficiency in plants is characterized by leaf necrosis, at least partly owing to a decrease in Mn-SOD levels accompanied by an increase in free oxygen radicals [8,81]. Manganese-SOD activity increases from the base to the tip in leaves, indicating that ROS production increases in peroxisomes or in the respiratory chain in mitochondria in aging mesophyll cells in wheat [82]. Transgenic tobacco plants carrying a unique Mn-SOD isoform targeted to the chloroplasts were shown to have a 1.5–2-fold higher Mn-SOD activity than untransformed plants [83]. This overexpression of Mn-SOD provided increased protection from oxidative stress caused by ROS generating chemicals, but did not provide protection from ROS generated via photooxidation under photoinhibitory conditions, most likely due to the sub-organellar localization of the enzyme [83,84].

## 7. Manganese-Dependent Oxalate Oxidase

Oxalate oxidase (OxOx) is a Mn-dependent germin enzyme, which catalyzes the oxygen dependent degradation of oxalate by oxidation into two moles of CO_2_, in a reaction that is coupled with the formation of H_2_O_2_ [85]. The involvement of Mn is unique, as there is no evidence for the involvement of other metals in the oxalate detoxification process [86]. Most knowledge on OxOx in plants has derived from work in cereals [86,87,88,89,90]. The activity of OxOx is localized to the apoplast and exerts a dual role in the defense of pathogens by destroying fungal toxins, and promoting lignification through the generation of H_2_O_2_ necessary for cross-linking reactions [91]. Oxalate oxidase is furthermore likely to have a role in seed maturation and germination, where isoforms of germin have been reported as discrete markers, however, the physiological relevance of the induction of OxOx activity during plant development is unclear [87].

Crystallographic assessment of OxOx from barley has revealed that Mn is bound by the side chains of a 3 His cluster and 1 Glu residue, as well as two adjacent water molecules in an octahedral (Oh, Figure 1a) metal complex [92]. The binding ligands are somewhat similar to those of Mn-SOD, with only Glu substituted with Asp (Figure 1b). The active site of OxOx is situated in the core of the complex composed of two nearly structurally equivalent β-barrel domains of the monomer, each containing a Mn ion. The active form of OxOx is a hexamer, organized as a trimer of dimers, in which each monomer has a canonical cupin fold [92,93]. Spectroscopic characterization has shown that the OxOx with the reduced Mn(II) form at the active center lacks activity, but the activity is restored when the metal center is oxidized to either Mn(III) or Mn(IV) forms. The proposed mechanism regulating the redox state of the metal center has been described by Whittaker et al. (2007) and involves the binding of oxalate as a monoanion directly to Mn center where an electron is transferred from the oxalate ligand to Mn(III). During the resulting reduction of Mn(III) to Mn(II) a highly unstable oxalyl free radical is formed, which undergoes spontaneous degradation to CO_2_ and a carbon dioxide radical anion (CO_2_^−^), which binds to the positive charge of Mn(II). In solution, CO_2_^−^ undergoes rapid electron transfer to dioxygen, yielding the protonated form of superoxide (the hydroperoxyl radical, HO_2_*) and an additional CO_2_. Hydroperoxyl exhibits a pKa of 4.8, meaning that this anionic form of the radical is by far the predominant species at physiological pH. Finally, the subsequent re-oxidation of Mn(II) to regain catalytic activity of OxOx occurs by electron transfer oxidation of Mn(II) by the hydroperoxyl radical to generate a molecule of H_2_O_2_.

Several pathogens are believed to make use of oxalate in pathogenesis, including *Sclerotinia sclerotiorum* (white mold) and *Erysiphaceae* (powdery mildew). The OxOx enzyme activity has been detected in young barley roots and in the leaves of mature plants in response to powdery mildew infection [94,95]. The response of the enzymes was detected already from 24 h after pathogen attack induced by inoculation [95]. The severity of take-all disease in wheat is related to the seed Mn content, with plants from seed with the largest Mn content yielding 165kg/ha more under severe disease conditions [96]. Given the requirement for Mn in OxOx, it is possible that resistance towards the disease is associated with OxOx activity, since the H_2_O_2_ produced by OxOx could be used in lignin cross-linking as described above. In support, two germin-like protein were identified in a GWAS mapping analysis of 248 barley varieties screening for candidate genes conferring tolerance to Mn deficiency [97].

As a means of protecting plants against the fungal toxin, the strategy of introducing oxalate degrading enzymes into plants, has been evaluated. When the OxOx gene from barley was introduced into oilseed rape it conferred resistance to exogenously supplied oxalic acid, as a result of increased levels of OxOx and enzyme activity [98]. In another study, transgenic oilseed rape constitutively expressing wheat OxOx showed increased OxOx enzyme activity and enhanced resistance towards *S. sclerotiorum* with up to 90% disease reduction compared to untransformed plants [99].

## 8. Conclusions

In this review we have used the EC classification of enzymes and the cofactor information contained in the UniProt Knowledgebase to provide an overview of the biochemical roles provided by Mn as an essential catalytic metal cofactor in plant enzymes. The distribution of Mn across all enzyme classes and its involvement in a large variety of enzymes is reflected by its multi-facetted chemistry, including redox state cycling, as well as an intriguing use of compartmentalization and chaperones to avoid mismetallation within plant cells. Knowledge on the role of Mn in biochemistry is accumulating, but the role of Mn in metalloenzyme activation and, not least, the resulting impact on metabolism is still in its infancy. A much better understanding of how metal homeostasis in the cell is regulated and how chaperones and metal sensors discriminate between elements is necessary to allow further understanding of the role of metals in the biochemistry of plants.

## Figures and Tables

**Table 1:** Chemical properties of selected divalent metal ions. The second ionization potential (eV) has proven to be a useful proxy for the polarizability of biologically relevant metal ions. The ionic radii are adapted from Irving and Williams [23] and all other values are adapted from Andreini et al. [24]. According to the hard and soft acids and bases (HSAB) classification, the metal ions (Lewis acids) are divided into hard (H), borderline (B), or hard-borderline (HB) types. The most common coordination geometries are abbreviated as follows: T_d_, tetrahedral; O_h_, octahedral; D_3h_, trigonal bipyramidal; D_4h_, square planar; C_4v_, square pyramidal; IR, irregular.

	Ca	Mg	Mn	Fe	Co	Ni	Cu	Zn
Ionic radius (Å) of the free metal ion	0.99	0.66	0.78	0.76	0.74	0.73	0.72	0.72
Second ionization potential (eV):	11.87	15.04	15.64	16.18	17.06	18.15	20.29	17.96
HSAB classification:	H	H	HB	B	B	B	B	B
Ligands:	O	O	N, O, S	N, S, O	N, O, S	S, N	S, N	N, S, O
Coordination numbers:	6,7,8	6	4, 5, 6	4,5,6	4,5,6	4,5,6	4,5,6	4,5,6
Dominating geometries:	O_h_, IR, IR	O_h_	T_d_, D_3h_, O_h_	T_d_/D_4h_, C_4v_/D_3H_, O_h_	T_d_, C_4v_/D_3h_, O_h_	O_h_, T_d_, D_4h_	T_d_/D_4h_, C_4v_/D_3h_, O_h_	T_d_, C_4v_/D_3h_, O_h_
